# Screening of Antioxidant and Antimicrobial Activity of *Micromeria fruticosa serpyllifolia* Volatile Oils: A Comparative Study of Plants Collected from Different Regions of West Bank, Palestine

**DOI:** 10.1155/2020/4851879

**Published:** 2020-07-15

**Authors:** Nihaya Salameh, Naser Shraim, Nidal Jaradat, Motasem El Masri, Lina Adwan, Shadi K'aibni, Raed Alkowni, Asma Radwan, Murad AbuAlhasan

**Affiliations:** ^1^Department of Pharmacy, Faculty of Medicine and Health Sciences, An-Najah National University, Nablus, P.O. Box 7, Nablus, State of Palestine; ^2^Faculty of Sciences, An-Najah National University, Nablus, P.O. Box 7, Nablus, State of Palestine; ^3^College of Pharmacy, Nursing and Health Professions, Birzeit University, P.O. Box 14, Birzeit, State of Palestine; ^4^Center of Birzeit University Testing Laboratories, Birzeit University, P.O. Box 14, Birzeit, State of Palestine

## Abstract

**Background:**

The investigation of volatile oils used in traditional medicine is vital to enhance the quality of healthcare. This study is aimed at screening the antioxidant and antimicrobial properties of *Micromeria fruticosa serpyllifolia* volatile oils from three different regions in Palestine (north, middle, and south).

**Methods:**

Volatile oils of three samples of *M. fruticosa serpyllifolia* were extracted using the microwave-ultrasonic apparatus. The antioxidant activity of the volatile oils was assessed by inhibition of DPPH free radical. The antimicrobial activity was examined using the broth microdilution method. Assessment of antifungal activity was achieved using the agar dilution method.

**Results:**

Screening the biological activity of plant extracts revealed that the sample from Ramallah (middle region) possessed the most potent antioxidant activity with an IC_50_ value of 0.45 *μ*g/mL. The three samples exhibited broad antimicrobial activity and showed potential antifungal activity. The sample from the southern region showed the highest potency against *Shigella sonnei* with the lowest reported MIC; the sample from the northern region demonstrated the least potency against clinical isolate of *Staphylococcus aureus* and “methicillin”-resistant *Staphylococcus aureus*.

**Conclusions:**

The study showed that *Micromeria fruticosa serpyllifolia* volatile oil samples from different regions in Palestine possess different potential antioxidant and antimicrobial activities that were in line with traditional uses of the plant extracts.

## 1. Background

Volatile oils (VOs) have antioxidant, anti-inflammatory, anticancer, anthelmintic, antimalarial, antiviral, antibacterial, cholesterol inhibition, and insecticide activities [1, 2]. The chemical structures of VOs determine their therapeutic activities [3]. Recently, there is great attention towards natural antioxidants from plants. Antioxidants can act as a radical scavenger, promote health, and produce anticancer activity [4].

There is a growing concern about antimicrobial resistance issue [5]. The complications of multidrug resistance enforced scientists to search for new antimicrobial agents from various sources such as medicinal plants [6]. In recent time, there has been considerable interest in VOs and extracts of medicinal plants for the development of alternatives to prevent or to delay the growth of pathogens [7]. Many scientific investigations reported that the chemical composition, total yield, and the aroma of VOs may be different due to growing conditions (climate, type of soil and composition, and altitude), plant age, geoclimatic location, and environmental conditions of collection time and site [8].

*Micromeria fruticosa* subspecies *serpyllifolia* (Lamiaceae), also known as White *Micromeria*, is an aromatic herb [9], dominant in the eastern Mediterranean regions including Palestine, which has a pleasant minty fragrance that in hot summer provides a sensation of coolness [10, 11]. In Palestinian society known as Duqat ‘Adas, ‘Ishbit esh-shai, Qurnya and as Thyme-leave savory in English, the aerial parts of the plant (flower, leaves, and stalk) are used in folk medicine [12]. It has been widely used in traditional medicine for the treatment of hypertension, heart disorders, diarrhea, abdominal pains, colds, headache, wounds, and infections such as skin and eye infections and as an anti-inflammatory agent [9, 11, 13–17]. *M. fruticosa serpyllifolia* VOs also exhibit antibacterial, antifungal, antioxidant, insecticide, analgesic, anticonvulsant, and CNS depressant activity [11, 15].

The authors of the current study conducted several tests on wild-growing *M. fruticosa serpyllifolia* in three regions in the West Bank area in Palestine. Part of these tests has been published previously [18]. Specifically, structural elucidation and identification of the chemical composition using the GC-MS analysis section represent shared results with this study, therefore elaborating and discussing some aspects linked to the findings of the published manuscript. As a continued step, the aims of this study were to screen antioxidant and antimicrobial activities of *M. fruticosa serpyllifolia* collected from three different regions of West Bank, Palestine.

## 2. Methods

### 2.1. Chemicals and Reagents

Calcium chloride, 2,2-diphenyl-1-picrylhydrazyl (DPPH), and Trolox were purchased from Sigma-Aldrich, Germany. Dimethyl sulfoxide (DMSO) was purchased from CARLO ERBA, France; Nutrient Agar, Mannitol, MacConKey Agar, and Mueller Hinton Broth were purchased from HiMedia Laboratories, Mumbai, India; Sabouraud Dextrose Agar was purchased from Oxoid, UK; 3-[N-morpholino] propanesulfonic acid (MOPS) buffer and RPMI-1640 medium (with L-glutamine, without sodium bicarbonate) (developed at Roswell Park Memorial Institute) were purchased from Sigma-Aldrich, UK. Tween 80 (0.05%) was purchased from ACROS Organics, Belgium. Sodium hydroxide and ethyl alcohol 99.9% were purchased from Sun Pharm. Drug Stars, Nablus, Palestine; cefuroxime 250 mg (as axetil) tablet and doxycycline 100 mg (hyclate) tablet (Jerusalem Pharmaceutical Company, Albiereh, Ramallah, Palestine), levofloxacin 500 mg tablet (Birzeit Pharmaceutical Company, Birzeit, Ramallah, Palestine), azithromycin 250 mg capsules (Pharmacare Company, Birzeit, Ramallah, Palestine), tinidazole 500 mg tablet (Jerusalem Pharmaceutical Company, Albiereh, Ramallah, Palestine), and terbinafine hydrochloride 250 mg tablet (Birzeit Pharmaceutical Company, Birzeit, Ramallah, Palestine), all these pharmaceuticals were donations from Military Medical Services Ramallah Palestine.

### 2.2. Instrumentation

Grinder (Moulinex model, Uno, China) was used to fracture the dried herbs. A balance (Radway ag, AS 220/c/2, Poland) was used to weigh the plant material; ultrasonic-microwave cooperative extractor/reactor (CW-2000, China) was employed for the extraction of volatile oil. A balance (Boeco, 4500 g, Germany), a UV-visible spectrophotometer (Jenway 7315, UK) for the assessment of the antioxidant activity of VOs, a water bath (Memmert, Germany), micropipettes (Finnpipette, Finland), a heater (Lab-Tech, Korea), and a balance (Sartorius AY 303, Canada) were used.

### 2.3. Plant Material Collection and Extraction Procedures

The aerial parts of *M. fruticosa serpyllifolia* were collected in April of 2017 from three cities in the West Bank (WB) in Palestine: Nablus, Ramallah, and Hebron representing north, middle, and south of the WB in Palestine, respectively. The samples were botanically identified and coded by Dr. Nidal Jaradat, the pharmacognosist at the Department of Pharmacy in Pharmacognosy and Herbal Products Laboratory, An-Najah National University, and the voucher specimen code was Pharm-PCT-1575. The extraction of VOs was performed following the procedure in accordance to [19, 20].

### 2.4. Antioxidant Activity

The scavenging activity of *M. fruticosa serpyllifolia* VOs of the three samples from three regions in WB in Palestine was assessed using the methods described in [20, 21]. Stock solutions at a concentration of 1 mg/mL in methanol and Trolox were prepared from *M. fruticosa serpyllifolia* VOs that were collected from three Palestinian regions. Each one of these stock solutions was diluted in methanol to prepare 12 of the working solutions with the following concentrations: 1, 2, 3, 5, 7, 10, 20, 30, 40, 50, 80, and 100 *μ*g/mL. A freshly prepared DPPH solution (0.002% *w*/*v*) was mixed with both methanol and with each of the abovementioned working solutions at 1 : 1 : 1 ratio. In addition, a negative control solution was prepared by mixing the mentioned DPPH solution with methanol in a 1 : 1 ratio. All of these solutions were incubated at room temperature in a dark cabinet for 30 min. By the end of the incubation period, the optical density of these solutions was determined using spectrophotometric absorbances at a wavelength of 517 nm using methanol as the blank solution.

Antioxidant activity was monitored by measuring the absorbance at 517 nm wavelength. The antioxidant activities of *M. fruticosa serpyllifolia* VOs and Trolox were assessed by their ability to donate a hydrogen atom or electron and were identified from converting the deep violet color of a methanol solution of DPPH to colorless or pale yellow; for that, the inhibition percentage of DPPH activity was used to determine the antioxidant activity of *M. fruticosa serpyllifolia* VOs and Trolox using the following equation (Inhibition% of antioxidant activity [6]):

(1) $$\begin{array}{c} \text{In}\%=\frac{A_{\text{blank}}-A_{\text{sample}}}{A_{\text{blank}}}\times 100, \end{array}$$

where *A*_blank_ represented the absorption of the control reaction (all reagent without the sample) and *A*_sample_ represented the absorbance of the sample.

### 2.5. Antimicrobial Screening

The antibacterial activities of *M. fruticosa serpyllifolia* VOs were investigated against the growth of nine reference bacterial strains obtained from the American Type Culture Collection (ATCC): *Escherichia coli* (ATCC 25922), *Enterococcus faecium* (ATCC 700221, USA), *Klebsiella pneumoniae* (ATCC 13883, UK), *Pseudomonas aeruginosa* (ATCC 27853, USA), *Shigella sonnei* (ATCC 25931, USA), and *Staphylococcus aureus* (ATCC 25923, USA). In addition, diagnostically proven clinical isolates *Proteus mirabilis*, *Staphylococcus aureus*, and methicillin-resistant *Staphylococcus aureus* (MRSA) were tested. Four antibiotics were used to test the sensitivity of bacterial strains: azithromycin, levofloxacin, cefuroxime, and doxycycline; the antibiotics were dissolved in a suitable solvent according to solubility test to obtain stock solution [22–24]. The antifungal activity of VOs was examined against the growth of two fungal strains acquired, from the American Type Culture Collection (ATCC), *Candida albicans* (ATCC 90028, USA) and *Epidermophyton floccosum* (ATCC 52066, UK). The antifungal agents (terbinafine and tinidazole) were used for susceptibility tests to *Candida albicans*, used with *M. fruticosa serpyllifolia* VOs [25]. The antibacterial activity of three VO samples was determined using broth microdilution method described in Jaradat et al. [20].

Each one of the isolated *M. fruticosa serpyllifolia* VOs was dissolved in DMSO (100%) at a concentration of 50 mg/mL. The prepared *M. fruticosa serpyllifolia* VO solutions were filter sterilized and then were serially microdiluted (2-fold) eleven times in sterile nutrient broth. In 96-well plates, the dilution processes were carried out under aseptic conditions. In the microwells that were assigned to evaluate the antibacterial activities of the extracted *M. fruticosa serpyllifolia* VOs, the concentration of these oils ranged from 0.024 to 25 mg/mL. On the other hand, the concentrations of these essential oils in the microwells assigned to evaluate their antifungal activities ranged from 8.467∗10^−3^ to 16.666 mg/mL. In these plates, microwell number 11 contained essential oil-free nutrient broth, which was used as a positive control for microbial growth. In addition, the microwell number 12 contained essential oil-free nutrient broth that was left uninoculated with any of the test microbes. This well was used as a negative control for microbial growth. Microwell numbers 1 to 11 were inoculated aseptically with the test microbes. At the time of inoculation, the final concentrations of microbial cells were about 2.5 × 10^5^ and 0.333–1.666 × 10^3^ colony-forming unit (CFU)/mL for the tested bacterial pathogens and *Candida albicans*, respectively. Each of the included microbes in this study was examined in duplicate for being inhibited by the *M. fruticosa serpyllifolia* essential oils. At 35°C, all the inoculated plates were incubated and the incubation period lasted for about 16-20 hours for the plates inoculated with the test bacterial strains and for about 48 hours for the plates inoculated with *Candida albicans*. The lowest concentration of *M. fruticosa serpyllifolia* essential oils, at which there was no visible microbial growth in that microwell, was observed.

### 2.6. Statistical Analysis

IC_50_ values of antioxidant activity and I% of DPPH free radical were determined in triplicate for *M. fruticosa serpyllifolia* VOs obtained from three different regions in Palestine. The results were expressed as mean ± standard deviation (SD), and the obtained data were compared using one-way ANOVA with post hoc Tukey-Kramer HSD multiple comparison calculation; *p* values of 0.05 or less were considered statistically significant [26].

## 3. Results

### 3.1. Yields and Chemical Composition

**Figure 1:**
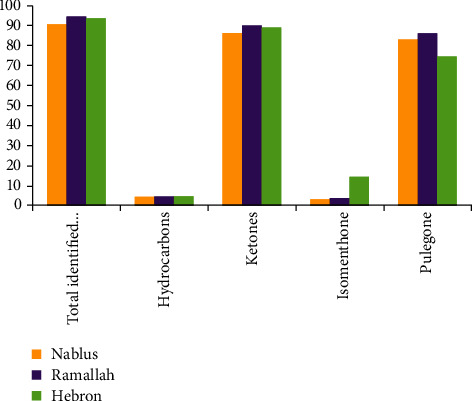
Chemical analysis of three samples of *M. fruticosa serpyllifolia* VOs.

Volatile oils of the three samples of *M. fruticosa serpyllifolia* were extracted using microwave-ultrasonic apparatus; still, the results of this section can be obtained from the previously published work by the same research group [18]. However, these results are shown in Figure 1 modified from [18]. Briefly, Figure 1 represents the chemical composition obtained using GC-MS analysis in which the most abundant components in all three samples were pulegone and isomenthone. The total identified components in the three samples were almost consistent in which 90.48, 94.44, and 93.55% of the constituents were identified in Nablus, Ramallah, and Hebron districts, respectively.

### 3.2. Antioxidant Activity

**Figure 2:**
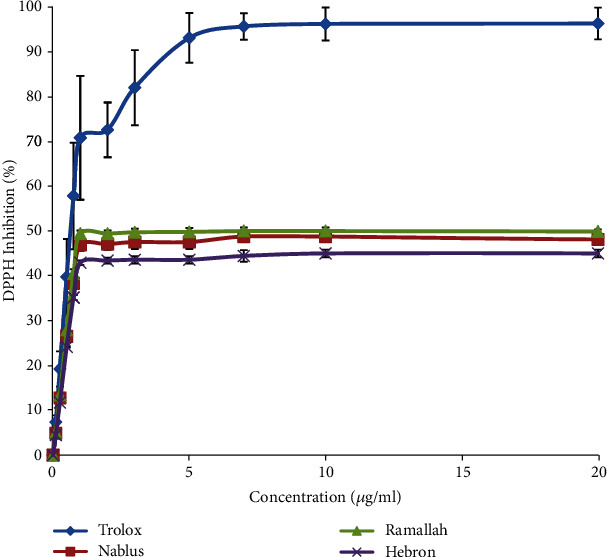
DPPH radical scavenging activity of the three samples of *M. fruticosa serpyllifolia* VOs and Trolox.

DPPH assay was used as in vitro approach to determine the free radical-scavenging activity and to screen for the possible antioxidant activity of the *M. fruticosa serpyllifolia* VOs from different regions in Palestine. IC_50_ values were used to assess the ability of the examined samples to inhibit DPPH. The assay revealed that the VO samples exhibited higher antioxidant potency compared to Trolox but they showed lower efficacy (maximum inhibition) (Table 1 and Figure 2). Statistical analysis using one-way ANOVA was performed to compare the antioxidant potency (IC_50_) and efficacy among samples. There were significant differences in antioxidant potency and efficacy of VOs compared to Trolox (*p* < 0.05 or <0.01). There were significant differences in antioxidant efficacy of VOs compared to each other (*p* < 0.05 or <0.01), but there were no significant differences in antioxidant potency of VOs compared to each other (*p* > 0.05).

### 3.3. Antimicrobial Activity

The minimum inhibitory concentrations (MICs) of *M. fruticosa serpyllifolia* VOs from different regions of Palestine were reported in Table 2. The majority of Gram (+) and Gram (-) bacterial strains were sensitive to *M. fruticosa serpyllifolia* VOs at MIC of 3.13 mg/mL. There were no statistically significant differences in activity against nine bacterial strains between *M. fruticosa serpyllifolia* VOs from the three regions in Palestine. There were significant differences of Hebron VO sample compared to Nablus and Ramallah VO samples against the American Type Culture Collection *Shigella sonnei* (ATCC 25931), *p* < 0.01, and the VO sample from Hebron had the highest potency at a MIC value of 1.56 mg/mL. There were significant differences in Hebron and Ramallah VO samples compared to Nablus VO samples against two clinical isolate of Gram (+) bacterial strains, *Staphylococcus aureus* and MRSA, *p* < 0.01, the VO sample from Nablus provided the lowest potency at a MIC value of 6.250 mg/mL. To evaluate the sensitivity of bacterial strains, four antibacterial drugs were used: azithromycin 250 mg, levofloxacin 500 mg, doxycycline 100 mg, and cefuroxime 250 mg. The MIC values of the drugs were in the range 1.28∗10^−6^ mg/mL–22.5∗10^−3^ mg/mL; Table 3 lists the MICs for drugs. In addition, the antifungal activity against the fungal strains was tested for sensitivity to *M. fruticosa serpyllifolia* VOs; the yeast was the most sensitive followed by the fungus; the American Type Culture Collection *C. albicans* (ATCC 90028) yeast was found to be the most sensitive to *M. fruticosa serpyllifolia* VO samples at a MIC value of 0.206 mg/mL followed by the fungus *Epidermophyton floccosum* (ATCC 52066) at a VO MIC value of 0.78 mg/mL (Table 2). To evaluate the sensitivity of fungal strains, two antifungal dugs were used: terbinafine 250 mg and tinidazole 500 mg, and the MIC value of antifungal drugs was 18.52 *μ*g/mL.

## 4. Discussion

Natural antioxidants have been widely investigated to find protective compounds against damages and diseases developed from free radicals and oxidative stress. *Micromeria* species were identified as a rich source of antioxidant agents [27]. Different results were reported by Güllüce and coauthors in Turkey in which the antioxidant activity of the VOs of *M. fruticosa serpyllifolia* was observed with an IC_50_ value of 98.2 *μ*g/mL [28]. The study showed that VOs abundant with oxygenated monoterpene such as pulegone have antioxidant activity [29, 30]. However, this result may support the antioxidant potency of Ramallah sample VOs, which contained the highest amount of total oxygenated compounds and pulegone (89.88 and 86.04%, respectively) among the three samples of VOs.

Interestingly, multidrug resistance of bacterial species causes health difficulties. Extracts of volatile oils have been investigated as new potential antimicrobial agents, biopreservative products, and promising antiseptic enhancers for topical uses [31]. *Micromeria* species VOs were considered to have strong broad-spectrum antimicrobial activity [32]. The results listed in Table 2 showed that the VOs of the three samples exhibited considerable antifungal potency but little antibacterial potency. The results of the antimicrobial activity of the VOs of three samples revealed that this activity was specific against *Shigella sonnei*, *Staphylococcus aureus* (CI), and MRSA and nonspecific against the rest of the microbial organisms. Screening the potential antimicrobial activity of *M. fruticosa serpyllifolia* VOs and methanolic extract, growing in Turkey conducted by Güllüce et al. [28], concluded that the VO provided stronger antimicrobial properties than methanolic extract (methanolic extract did not show any antimicrobial activity); the MIC values of 0.5 mg/mL volatile oil stock solution for bacterial species which were susceptible to the oil ranged from 31.25 to 125 *μ*g/mL and for fungi which were susceptible to VO ranged from 31.25 to 62.50 *μ*g/mL. A study conducted by Omari et al. [33] evaluating the antifungal activity of *M. barbata* growing in Lebanon, using different fungal strains and yeasts, including *Epidermophyton floccosum* and *Candida albicans*, concluded that the *M. barbata* VOs showed a high fungistatic activity. Investigating the antimicrobial activity of *Micromeria cilicica* VOs growing in Turkey resulted in the finding that the *Micromeria cilicica* VOs and pulegone crude compound (the main component) showed a significant antifungal and antibacterial activity; the activities increased relying on the amount of pulegone and VOs and *Candida albicans* was the most sensitive to pulegone [34]. *Micromeria congesta* VOs were considered as a significant antibacterial due to abundant components such as pulegone and isomenthone [35]. Studying the chemical ingredients and antibacterial and antifungal activity of the volatile oils of four plants including *Mentha spicata* growing in Iran by Kazemi et al. [36], and crude menthone (the dominant component) for antimicrobial activity, reported that VOs showed very strong antimicrobial properties against *Staphylococcus aureus*, all of *Shigella species*, *Escherichia coli*, *Klebsiella sp.*, *Pseudomonas aeruginosa*, *Proteus sp.*, *Candida albicans*, and other strains and concluded that menthone (isomenthone) exhibited strong antibacterial properties with MIC 1.5-3.5 *μ*g/mL. These findings could be linked with our results obtained from Hebron in which the VOs of *M. fruticosa serpyllifolia* owned the highest quantity of isomenthone (14.41%) which is thought to be effective against *Shigella sonnei*. On the contrary, isomenthone represented the lowest amount of constituents (3.16%) in Nablus and therefore exhibited the lowest potency against *Staphylococcus aureus* (CI) and MRSA.

## 5. Conclusions

*M. fruticosa serpyllifolia* VOs from different regions in Palestine represented by three cities showed variable antioxidant and antimicrobial activities depending on the phytochemical constituents of the volatile oils. The sample from middle Palestine (Ramallah) showed the most potent antioxidant properties. The plant extract exhibited strong antifungal activities and minimal antibacterial activities. The sample of the south region showed higher potency against *Shigella sonnei* while the sample of the northern region showed lower potency against *Staphylococcus aureus* (CI) and MRSA. These findings enable *M. fruticosa serpyllifolia* VOs to be good agents in curing or preventing oxidative stress and healing wounds and skin dermatitis and a good food preservative agent.

Further *in vivo* studies are needed to evaluate the potential pharmacological activities, to isolate the basic components responsible for potential pharmacological activities, and to evaluate the safety and toxicity of plant extract.

## Figures and Tables

**Table 1:** IC_50_ of DPPH radical scavenging activity of *M. fruticosa serpyllifolia* VOs from different regions of Palestine and Trolox.

	Trolox	Nablus	Ramallah	Hebron
IC_50_ (*μ*g/mL)	0.64 ± 0.12	0.47 ± 0.02^a^	0.45 ± 0.01^a^	0.47 ± 0.01^d^
Max. I% DPPH radical scavenging activity	96.80 ± 2.83	49.25 ± 0.33^d^	50.19 ± 0.65^bd^	45.01 ± 0.86^cde^

^a^ *p* < 0.05 compared to Trolox, ^b^*p* < 0.05 compared to Nablus, ^c^*p* < 0.01 compared to Ramallah, ^d^*p* < 0.01 compared to Trolox, and ^e^*p* < 0.01 compared to Nablus; ^∗^mean ± SD, *n* = 3.

**Table 2:** Antimicrobial activity (MIC in mg/mL) of *M. fruticosa serpyllifolia* VOs from different regions of Palestine based on the broth microdilution method and agar dilution method.

	MIC Nablus	MIC Ramallah	MIC Hebron	DMSO 100%
Yeast				
*C. albicans* (ATCC 90028)	0.206	0.206	0.206	3.70%
Fungus				
*Epidermophyton floccosum* (ATCC 52066)	0.781	0.781	0.781	6.25%
Bacterial strains				
*Staphylococcus aureus* (ATCC 25923)	3.125	3.125	3.125	12.50%
*Staphylococcus aureus* (CI)	6.250	3.125^a^	3.125^a^	12.50%
MRSA (CI)	6.250	3.125^a^	3.125^a^	12.50%
*Enterococcus faecium* (ATCC 700221)	3.125	3.125	3.125	6.25%
*Escherichia coli* (ATCC 25922)	3.125	3.125	3.125	12.50%
*Pseudomonas aeruginosa* (ATCC 27853)	3.125	3.125	3.125	12.50%
*Shigella sonnei* (ATCC 25931)	3.125	3.125	1.5625^ab^	12.50%
*Proteus mirabilis* (CI)	3.125	3.125	3.125	12.50%
*Klebsiella pneumoniae* (ATCC 13883)	3.125	3.125	3.125	12.50%

^a^ *p* < 0.01 compared to Nablus, ^b^*p* < 0.01 compared to Ramallah.

**Table 3:** Minimum inhibitory concentration (*μ*g/mL) of some antimicrobial drugs.

	Azithromycin	Levofloxacin	Doxycycline	Cefuroxime
Bacterial strains				
*Staphylococcus aureus* (ATCC 25923)	0.352	5.125∗10^−3^	0.012	2.356
*Staphylococcus aureus* (CI)	0.352	6.4∗10^−3^	0.097	4.713
MRSA (CI)	0.176	6.4∗10^−3^	0.097	4.713
*Proteus mirabilis* (CI)	5.625	1.28∗10^−3^	0.387	4.713
*Pseudomonas aeruginosa* (ATCC 27853)	0.703	1.28∗10^−3^	0.387	2.356
*Escherichia coli* (ATCC 25922)	0.703	1.28∗10^−3^	0.012	2.356
*Klebsiella pneumoniae* (ATCC 13883)	1.406	0.012	0.387	4.713
*Shigella sonnei* (ATCC 25931)	0.703	—	0.387	2.356
*Enterococcus faecium* (ATCC 700221)	22.5	1.64	0.0.097	4.713
	Terbinafine	Tinidazole		
*Candida albicans* (ATCC 90028)	18.5185	—		

## Data Availability

All raw data are available upon request from the corresponding author.

## Abbreviations

CNS: Central nervous system
DMSO: Dimethyl sulfoxide
DPPH: 2,2-Diphenyl-1-picrylhydrazyl
GC-MS: Gas chromatography mass spectrometry
MIC: Minimum inhibitory concentration
MRSA: Methicillin-resistant *Staphylococcus aureus*
VOs: Volatile oils.

## Supplementary Materials

Supplementary Materials Supplementary data 1: GC-MS chromatograms of volatile oil samples of *M. fruticosa serpyllifolia* collected from different regions. Supplementary data 2: GC-MS analysis and component identification and quantification of volatile oil samples. Supplementary data 3: detailed procedures for antibacterial agent preparation. Supplementary data 4: detailed protocol for antifungal agent preparation. Supplementary data 5: antimicrobial effect of *M. fruticosa serpyllifolia* volatile oils collected from different regions using the agar dilution method.
