# Supplementary material for: Screening of Antioxidant and Antimicrobial Activity of Micromeria fruticosa serpyllifolia Volatile Oils: A Comparative Study of Plants Collected from Different Regions of West Bank, Palestine
Source: Biomed Res Int. 2020 Jul 15;2020:4851879. doi: 10.1155/2020/4851879 (PMC7378623; doi:10.1155/2020/4851879)
Supplement: Supplementary Materials — Supplementary data 1: GC-MS chromatograms of volatile oil samples of M. fruticosa serpyllifolia collected from different regions. Supplementary data 2: GC-MS analysis and component identification and quantification of volatile oil samples. Supplementary data 3: detailed procedures for antibacterial agent preparation. Supplementary data 4: detailed protocol for antifungal agent preparation. Supplementary data 5: antimicrobial effect of M. fruticosa serpyllifolia volatile oils collected from different regions using the agar dilution method. [file 4851879.f1.docx]

# Supplementary data 1


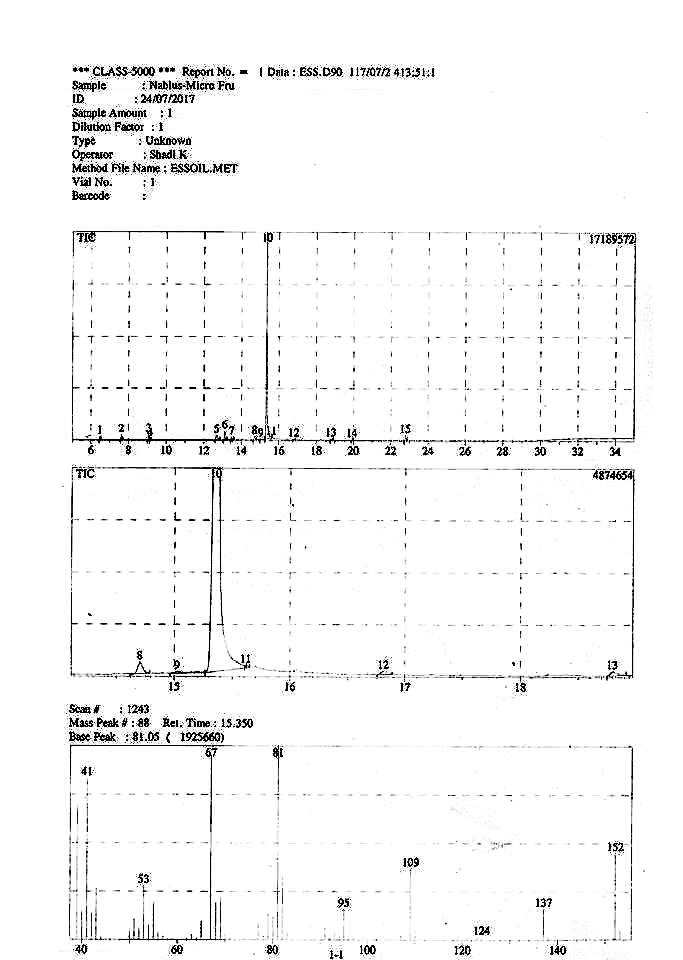


Chromatogram *of M fruticosa serpyllifolia* VO of Nablus


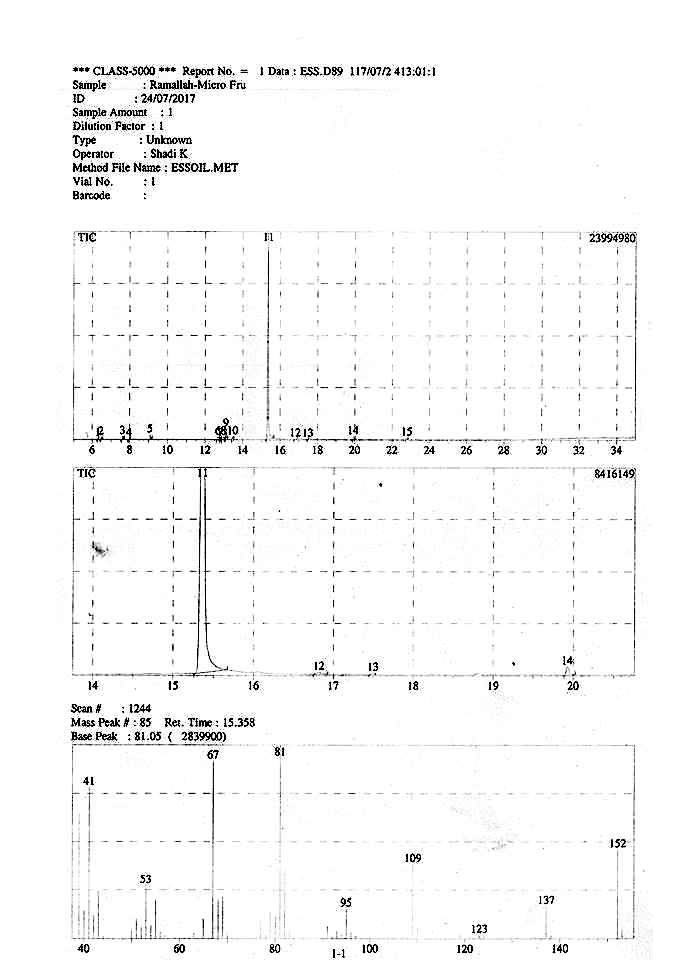


Chromatogram of *M fruticosa serpyllifolia* VO of Ramallah


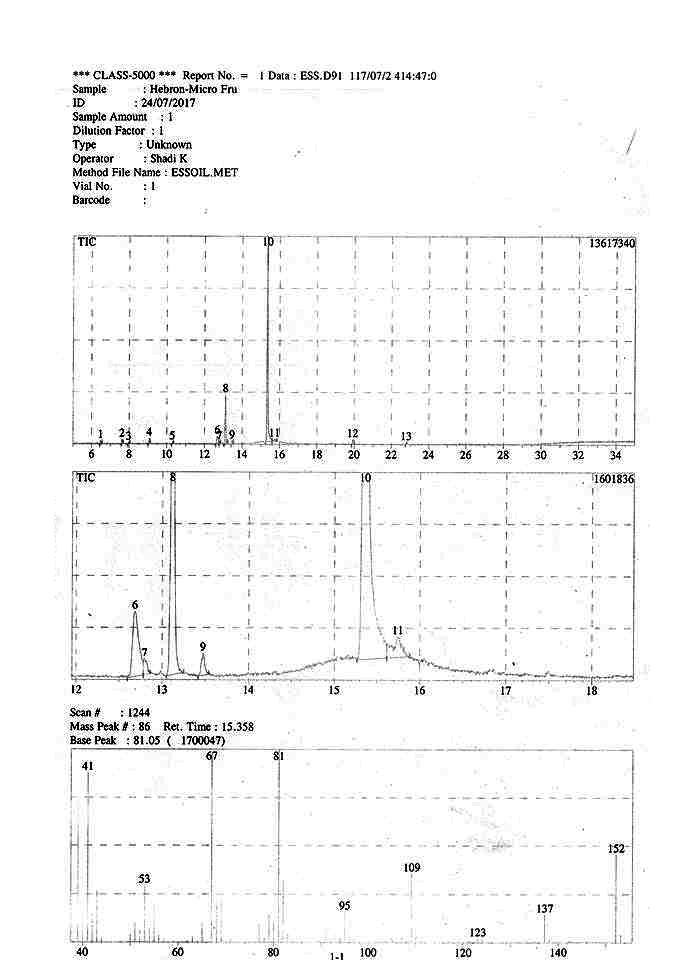


Chromatogram of *M fruticosa serpyllifolia* VO of Hebron

Supplementary data 2


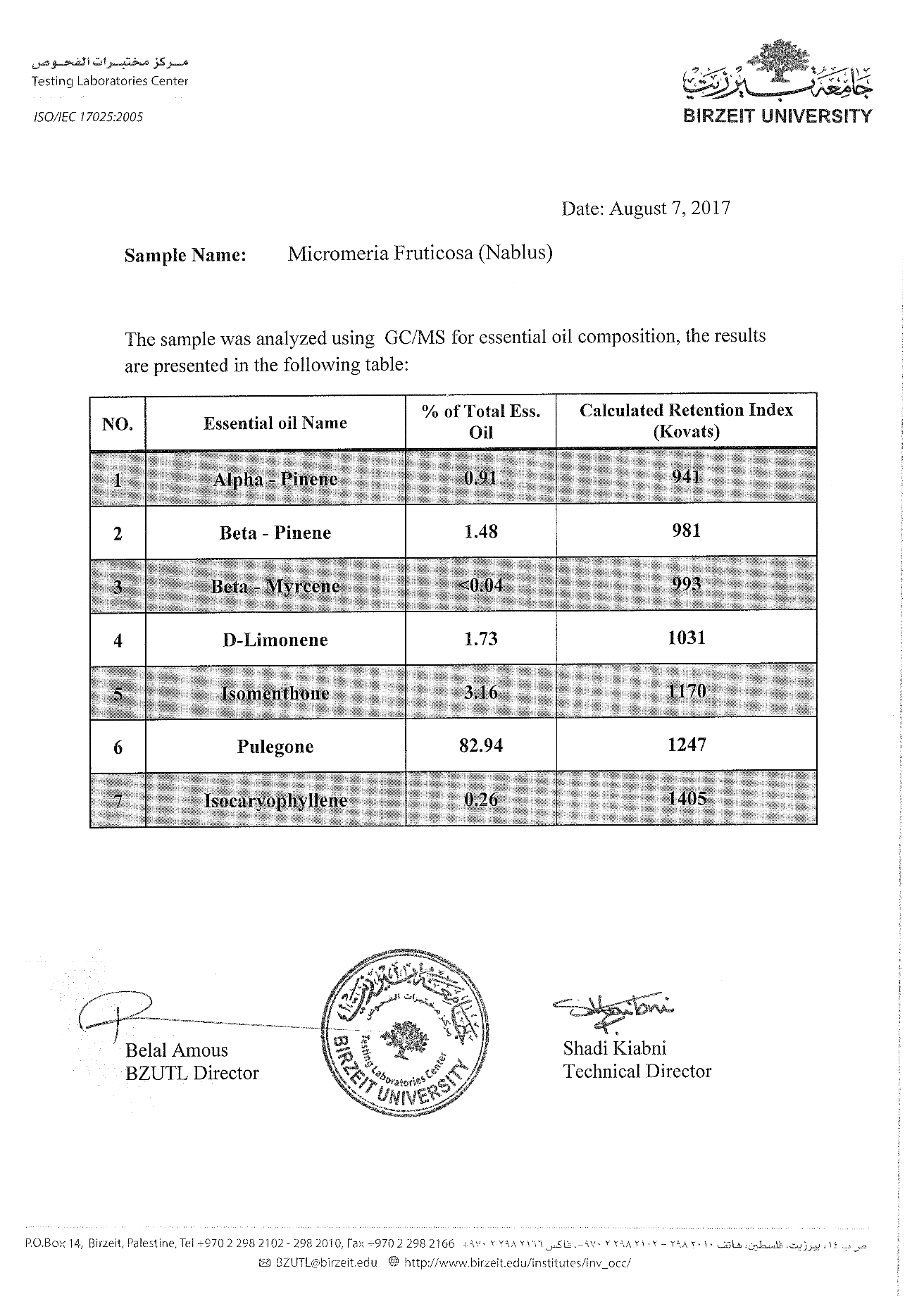


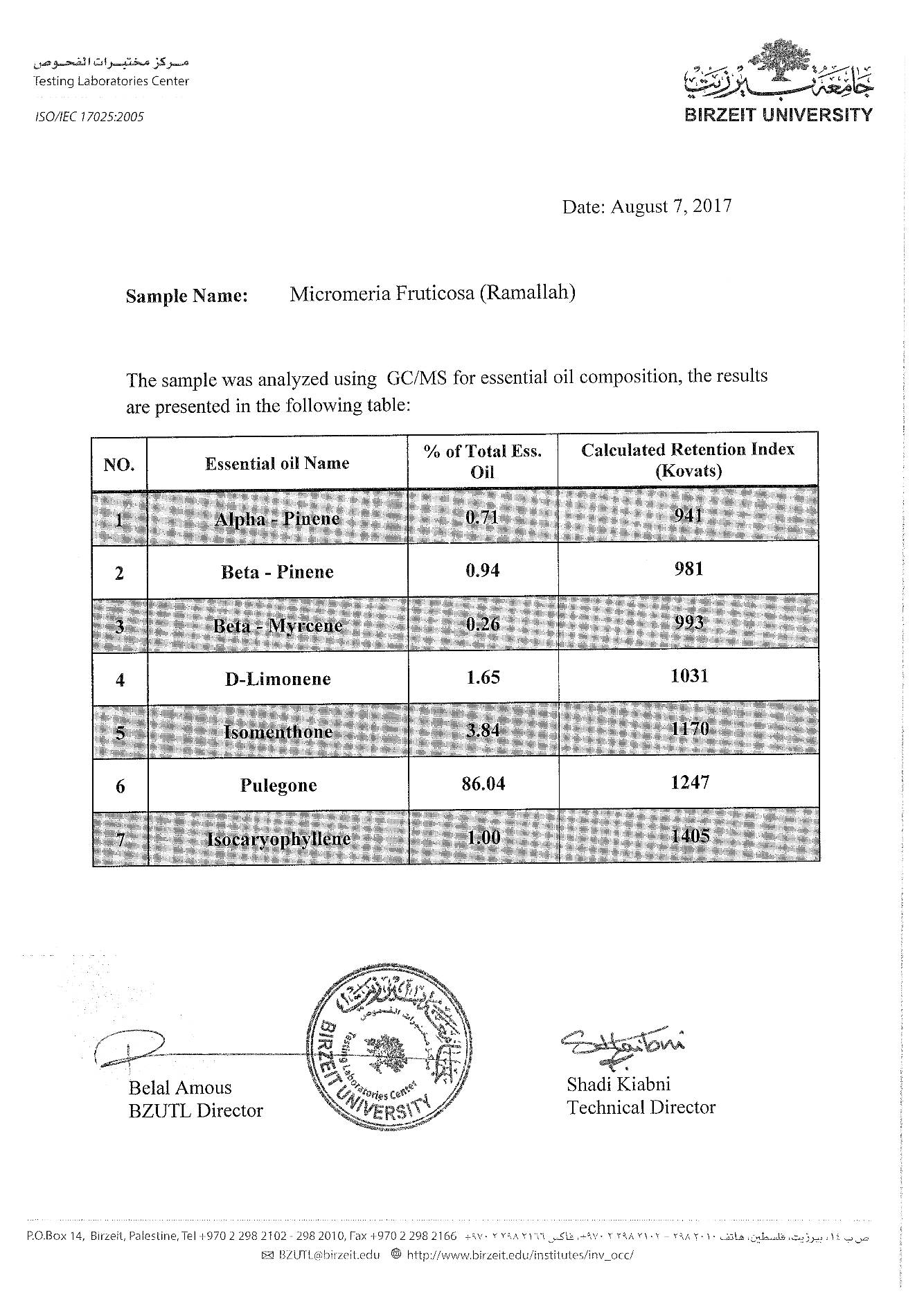


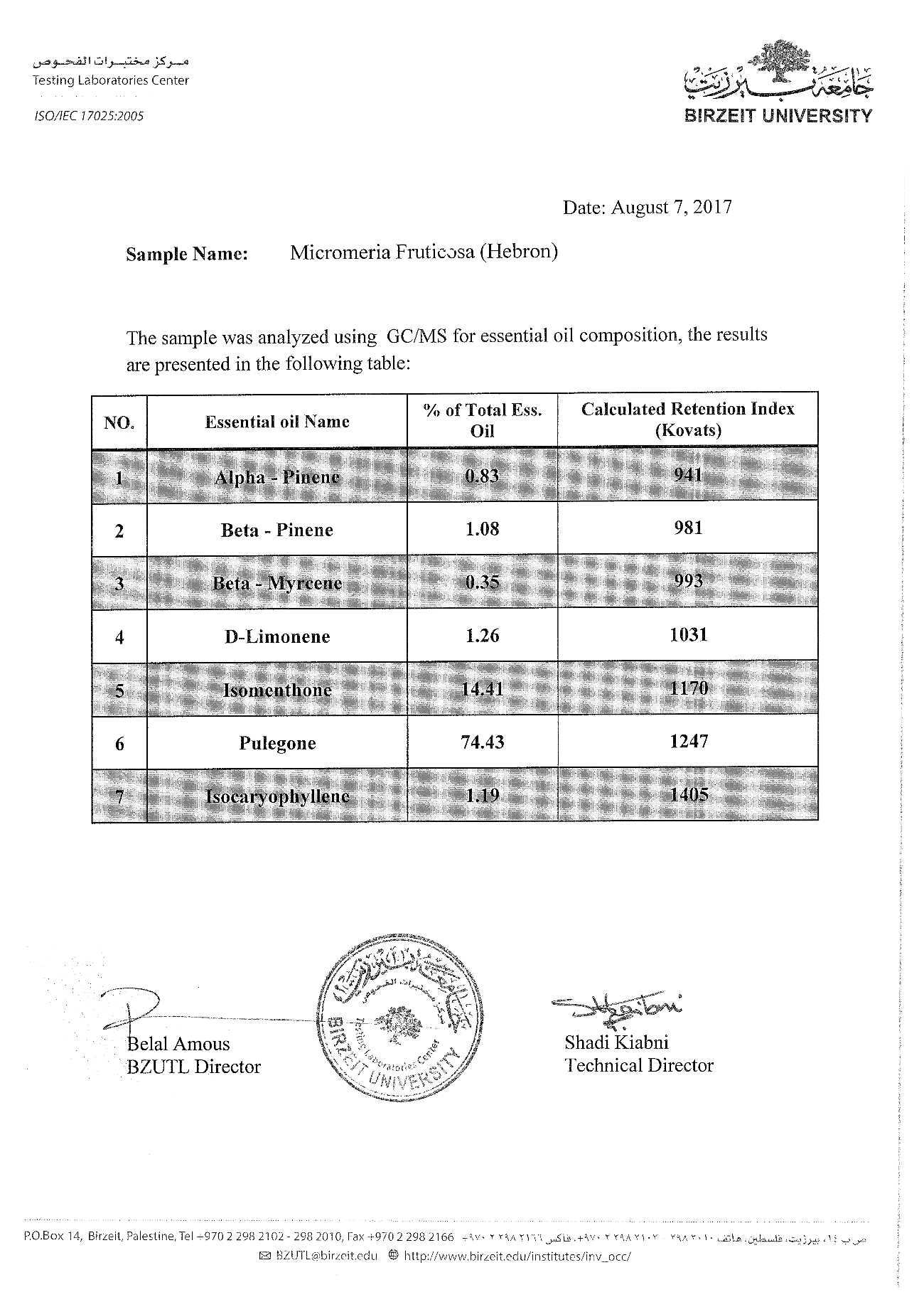


Supplementary data 3

**Preparation of Antibacterial agents:**

**Azithromycin** was dissolved in Ethanol 95% to get stock solution of concentration (4.5 mg/mL), two dilutions of (1:10) were made to get concentration (45µg/mL) carried out on all bacterial strains. **Levofloxacillin** was dissolved in sterile distilled water to acquire stock solution of concentration (3.28 mg/mL), three dilutions of (1:10) were made to get concentration (3.28 µg/mL), was performed on *Staphylococcal enterotoxin* B (SEB), MRSA, *Enterococcus faecium* and *Klebsilla pneumoniae*, and a fourth dilution was made of (1:10) to get concentration (0.328 µg/mL) was performed on *Staphylococcus aureus*, *Proteus mirabilis*, *Pseudomonas aeruginosa*, *Escherichia coli* and *Shigella sonnie*. **Cefuroxime** was dissolved in Dimethyl sulfoxide (DMSO) 100% to obtain a stock solution of concentration (3.77 mg/mL), two dilutions of (1:10) were made to get concentration (37.7 µg/mL), was tested on all bacterial strains. **Doxycycline** was dissolved in DMSO 100% to get a stock solution of concentration (6.20 mg/mL), three dilutions were made of (1:10) to get concentration of (6.20 µg/mL), was carried out on all bacterial strains

Supplementary data 4

**Preparation of Antifungal agents**

**Terbinafine** was dissolved in equal portions of sterile distilled water and methanol (1:1) to get a stock solution of concentration (50 mg/mL), two dilutions were made of (1:10) to obtain concentration of (500 µg/mL). **Tinidazole** was dissolved sterile distilled water to get a stock solution of concentration of (50 mg/mL), two dilutions were made of (1:10) to obtain concentration of (500 µg/mL).

Supplementary data 5


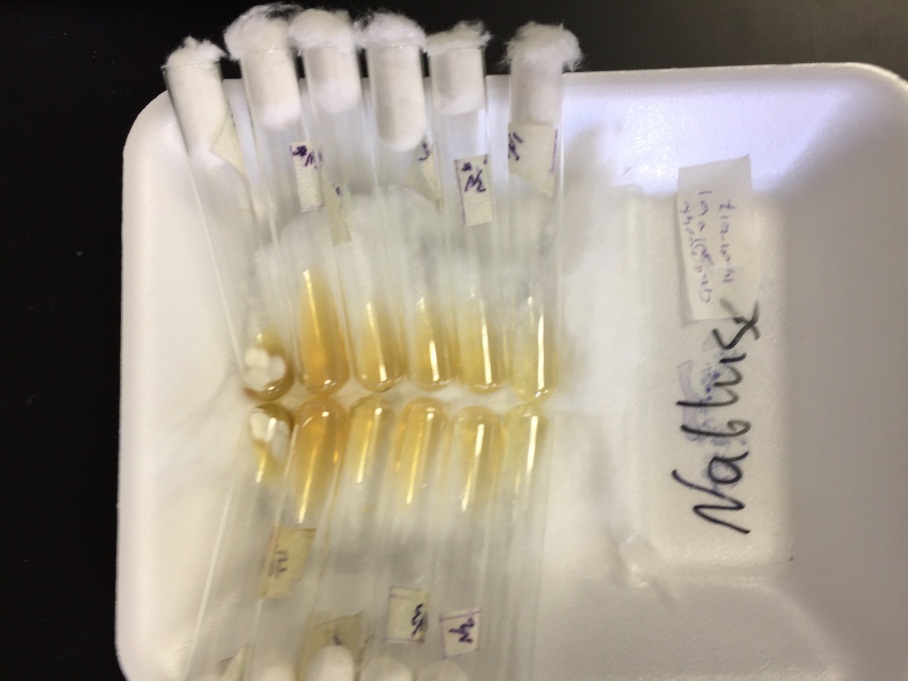


Effect of *M fruticosa serpyllifolia* VOs from Nablus on *Epedermophyton floccosum* using Agar dilution method


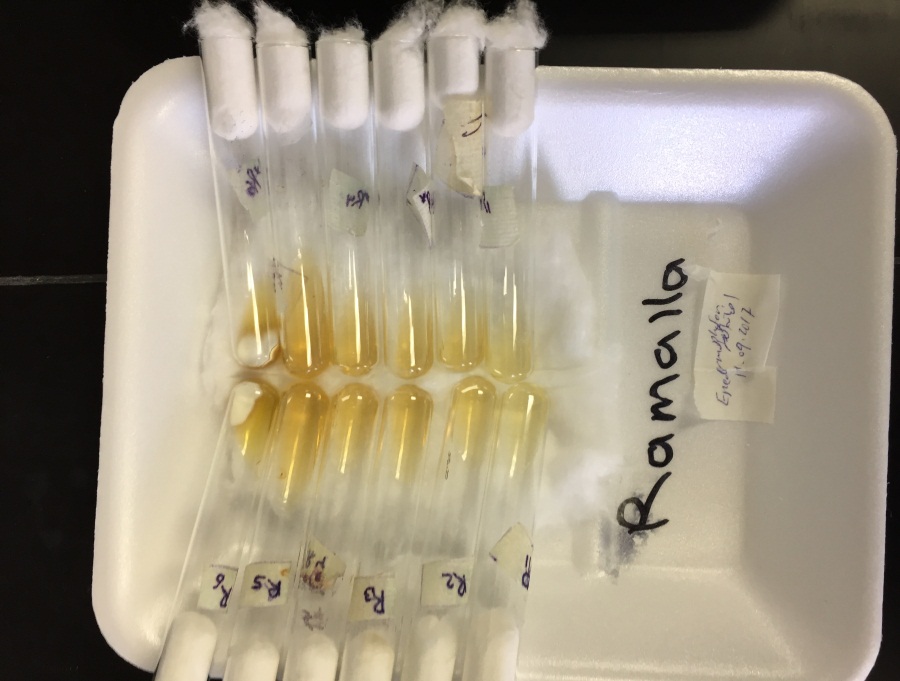


Effect of *M fruticosa serpyllifolia* VOs from Ramallah on *Epedermophyton floccosum* using Agar dilution method

# Appendix 10


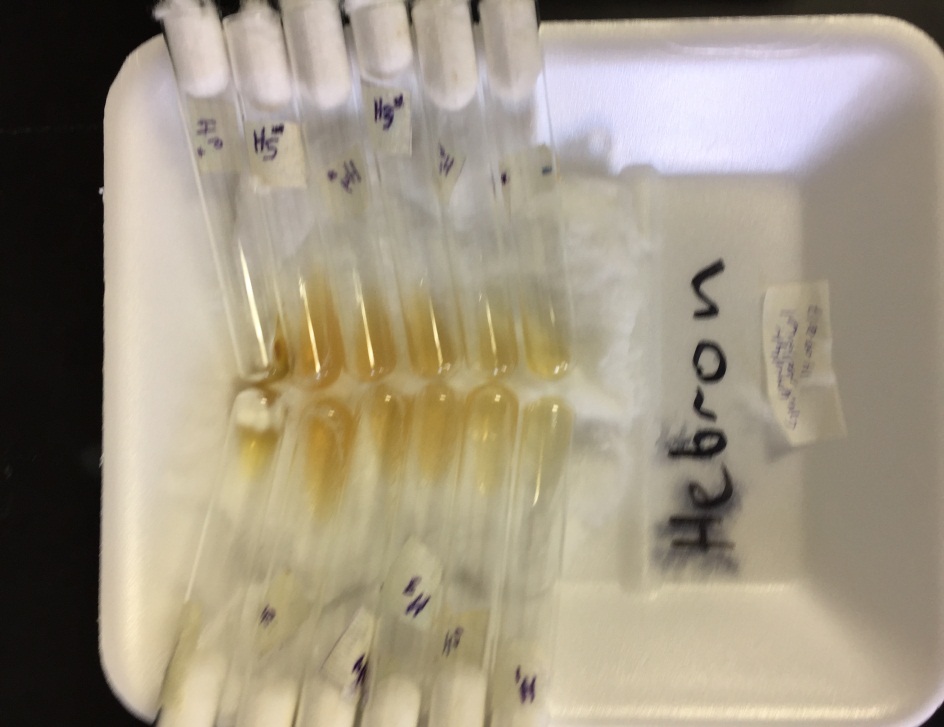


Effect of *M fruticosa serpyllifolia* VOs from Hebron on *Epedermophyton floccosum* using Agar dilution method


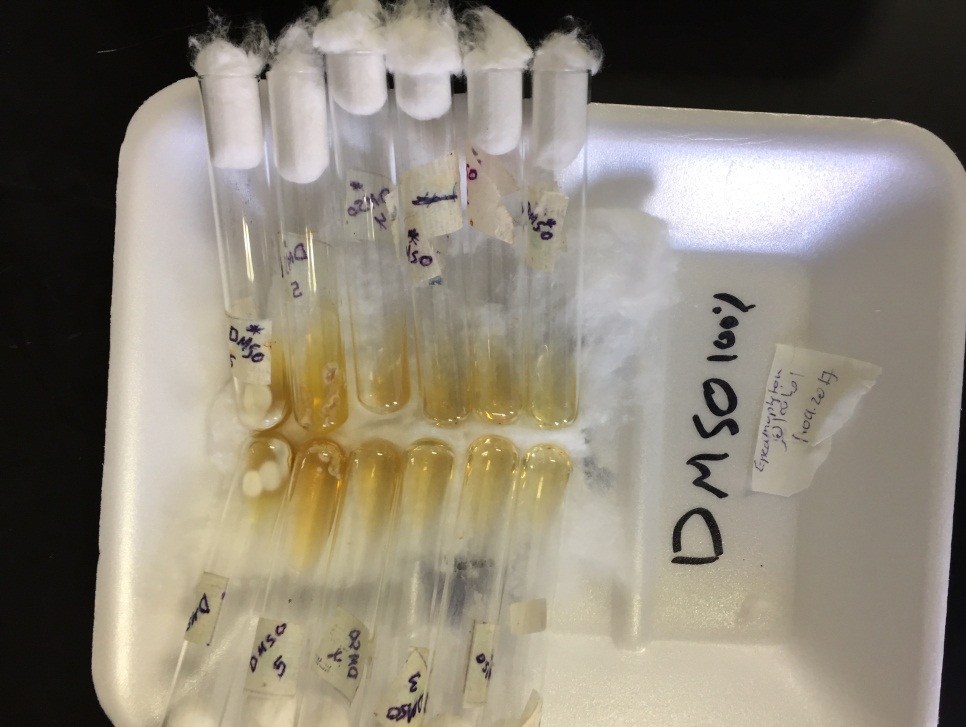


Effect of *M fruticosa serpyllifolia* VOs from DMSO 100% on *Epedermophyton floccosum* using Agar dilution method
